# Protein–Protein Interactions in Translesion Synthesis

**DOI:** 10.3390/molecules26185544

**Published:** 2021-09-13

**Authors:** Radha Charan Dash, Kyle Hadden

**Affiliations:** Department of Pharmaceutical Sciences, University of Connecticut, 69 North Eagleville Rd, Storrs, CT 06029-3092, USA; radha.dash@uconn.edu

**Keywords:** protein–protein interaction, translesion synthesis, cancer, REV1, polymerase ζ

## Abstract

Translesion synthesis (TLS) is an error-prone DNA damage tolerance mechanism used by actively replicating cells to copy past DNA lesions and extend the primer strand. TLS ensures that cells continue replication in the presence of damaged DNA bases, albeit at the expense of an increased mutation rate. Recent studies have demonstrated a clear role for TLS in rescuing cancer cells treated with first-line genotoxic agents by allowing them to replicate and survive in the presence of chemotherapy-induced DNA lesions. The importance of TLS in both the initial response to chemotherapy and the long-term development of acquired resistance has allowed it to emerge as an interesting target for small molecule drug discovery. Proper TLS function is a complicated process involving a heteroprotein complex that mediates multiple attachment and switching steps through several protein–protein interactions (PPIs). In this review, we briefly describe the importance of TLS in cancer and provide an in-depth analysis of key TLS PPIs, focusing on key structural features at the PPI interface while also exploring the potential druggability of each key PPI.

## 1. Introduction

DNA damage is an abnormal change in the basic structure of DNA that can be caused by external agents such as sunlight, ionizing radiation, chemotherapy as well as intracellular metabolism [1]. Cells respond to DNA damage by initiating DNA damage repair (DDR) mechanisms that eliminate lesions and restore the original DNA sequence [2,3]. Despite the presence of highly efficient DDR mechanisms, some DNA lesions may escape repair and interfere with the progression of replication forks [3]. These escaped DNA lesions may cause arrest of replication forks and the generation of post-replication gaps [4,5]. In this scenario, cells use DNA damage tolerance (DDT) mechanisms to bypass lesions encountered during active replication. There are two primary DDT mechanisms through which this DNA damage is bypassed to allow replication to continue. The first, termed translesion synthesis (TLS), involves the replicative bypass of the lesion by error-prone DNA TLS polymerases [6]. The other DDT mechanism, template switching (TS), involves the stalled nascent strand temporarily switching to the newly synthesized undamaged sister strand for replication past the lesion [7]. Most commonly, TLS and TS are utilized for single-strand DNA (ssDNA) legion lesion repair and homology-dependent repair of double-strand breaks (DSBs). Each of these mechanisms enables bypass of the lesion and completion of replication, without removing or repairing the lesion. Thus DDT is not a repair pathway, but provides a mechanism for tolerating DNA lesions during replication, thereby increasing survival and preventing genome instability. As TLS DNA polymerases lack proofreading activity and have large active sites that can recognize modified nucleotides, they are error-prone and commonly incorporate the non-Watson–Crick base pairs, which can potentiate the mutagenic nature of the cell. By contrast, TS is an error-free process [8,9].

In mammalian cells, TLS-mediated replication occurs past a diverse array of DNA lesions by the sequential action of several TLS DNA polymerases (POLs) in a heteroprotein complex that also includes the DNA sliding clamp proliferating cell nuclear antigen (PCNA). These include the B-family polymerase POLζ (complex of REV3/REV7/POLD2/POLD3), and the four Y-family polymerases, REV1, POLη, POLι and POLκ [10]. Two A-family polymerases, POLθ [11] and POLν [12], have also been implicated in TLS, but a clear role for either has not been conclusively defined [13]. The Y-family POLs are known as “inserter” polymerases because their primary role is insertion of a nucleotide across from the damaged DNA. POLζ acts to extend the primer template past the inserted nucleotide and is commonly referred to as the “extender” polymerase.

**Figure 1:**
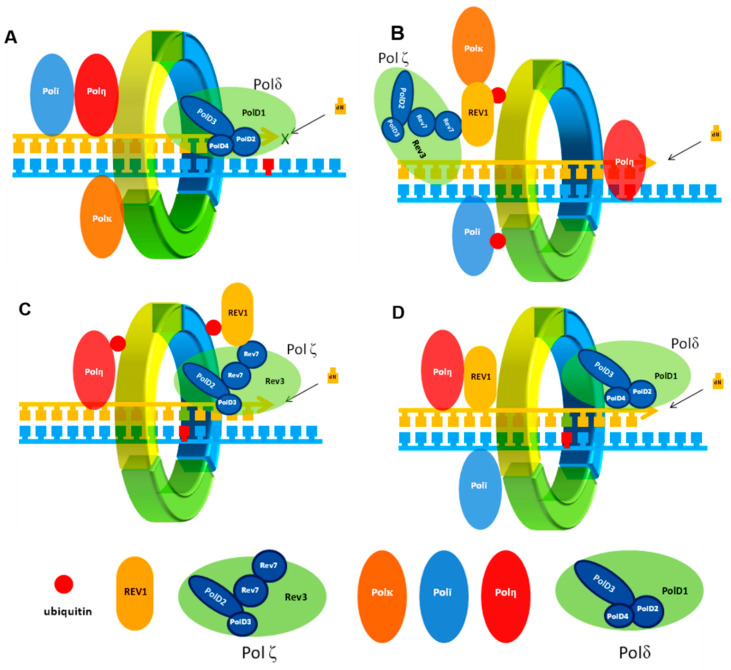
Basic mechanism of TLS in eukaryotic cells. (**A**) Stalling of the replicative DNA POL (here shown as POLδ, consisting of POLD1, POLD2, POLD3, and POLD4 subunits) at a damaged site (red). (**B**) PCNA ubiquitination recruits REV1 and other DNA TLS polymerases to the primer terminus (here shown for POLζ) and nucleotide (mis)insertion opposite the lesion. (**C**) Extension (catalyzed by POLζ) of the resulting primer termini by several nucleotides. (**D**) De-ubiquitination of PCNA and release of the TLS polymerase after bypass completion allowing the normal replication restart.

Because REV1/POLζ-dependent TLS is inherently mutagenic, a complicated multi-step process is employed to ensure that TLS polymerases are only recruited to DNA lesions during active replication (Figure 1). When a B-family replicative polymerase such as POLδ encounters DNA damage, it triggers a cascade of events that initiate the TLS mechanism. The stalling of POLδ on the DNA lesion leads to monoubiquitination of the DNA sliding clamp proliferating cell nuclear antigen (PCNA) on K164 by the E3 ubiquitin ligase Rad6/Rad18 at the damaged strand. The ubiquitination of PCNA (UbPCNA) serves to localize REV1 to stalled replication forks, which in turn recruits an inserter polymerase to initiate the TLS catalytic process. REV1 interacts with UbPCNA via its ubiquitin-binding domain, which is composed of two tandem ubiquitin-binding motifs (UBM1 and UBM2) [14,15,16,17]. Recent studies suggest that UBM2 is the primary site for the UbPCNA/REV1 protein–protein interactions (PPI) responsible for REV1-mediated DNA damage tolerance [18,19]. REV1 was originally identified as an adeoxycytidyl transferase that inserts dC nucleotides irrespective of what is on the template strand; however, subsequent studies have demonstrated that its major role in TLS is as a scaffolding protein to organize the various TLS components for DDT. The ubiquitination step is followed by the insertion step where the C-terminal domain of REV1 (REV1-CT) recruits the necessary inserter polymerase to the site of the DNA damage. The Y-family TLS DNA polymerases POLη, POLι, POLκ are considered the primary inserter polymerases because their activity is limited to the incorporation of one or a few nucleotides opposite the damaged site. These inserter polymerases differ in their sequence similarities, efficiency, accuracy, and the spectrum of nucleotides they incorporate opposite the lesion. POLη, POLι and POLκ can also function independently of REV1 by directly interacting with PCNA via the UBM domain and/or the PCNA interacting protein (PIP)-box domain [20,21,22]. For the insertion step, interactions of REV1 with its protein partners are critically dependent on its C-terminal domain [23,24], which interacts with the REV1-interacting-region (RIR) of its partner proteins. Insertion of the nucleotide opposite the lesion is followed by the elongation step of the resulting 3′ terminus by the extender TLS DNA polymerase POLζ. Again, the REV1-CT domain plays a crucial role in extender polymerase recruitment through PPIs with the POLζ subunit REV7 (Figure 1C). Finally, deubiquitination and/or ISGylation of PCNA results in release of the error-prone TLS POLs after bypass completion, allowing normal replication to restart [25]. These mechanistic details of REV1/POLζ-dependent TLS are depicted in Figure 1.

As TLS is an important DDT mechanism essential for proper cell survival and genome maintenance, dysregulation of TLS function can play an etiological role in the formation of cancer. Aberrant regulation or changes in expression of TLS POLs has been linked to cancer formation in a variety of different tissues [26,27,28]. From a therapeutic standpoint, TLS has also been implicated as an essential mechanism through which cancer cells develop resistance to multiple anti-cancer drugs. TLS-mediated bypass of chemotherapeutic-induced DNA lesions in cancer cells compromises the therapeutic efficacy of several first-line genotoxic agents. The primary mechanism of action for these drugs (platinating agents, alkylating agents, temozolomide, etc.) is direct chemical modifications to DNA, which ultimately leads to cell death. TLS in cancer cells can bypass these lesions, preventing apoptosis and resulting in a surviving population of tumor cells with increased mutations and a greater potential to develop acquired resistance to the first-line agent(s). Disruption of REV1/POLζ-dependent TLS in a variety of cancer models restores sensitivity to several genotoxic agents, reduces tumor progression, and can increase overall survival. More details about the role of TLS in the onset and progression of cancer, as well as its role in the development of acquired resistance to genotoxic agents can be found in several recent review articles [26,27,28].

## 2. Protein–Protein Interactions (PPIs) in TLS

**Figure 2:**
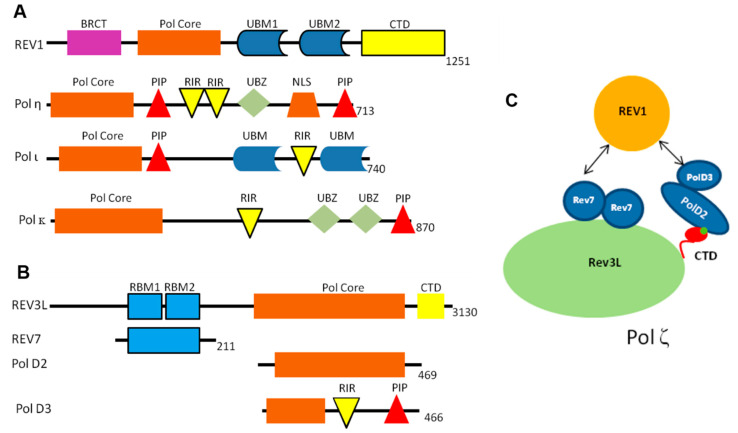
Proteins involved in TLS. Schematic illustration of the domain structures of Y-family POLs (**A**) and the multi-subunit TLS enzyme POLζ (**B**). (**C**) Schematic of human POLζ. A dimer of REV7 binds to the catalytic subunit of POLζ, REV3L. POLD2 binds REV3L at the CTD, coordinated by the iron-sulfur (4Fe-4S) cluster. REV1 binds human POLζ through interactions with REV7 and POLD3. BRCT, BRCA C-terminal domain; CTD, C-terminal domain; NLS, nuclear localization signal; PIP, PCNA-interacting peptide; RIR, REV1-interacting region; UBM, ubiquitin-binding motif; and UBZ, ubiquitin-binding domain.

As TLS involves multiple proteins coming together in a complicated and precise process, the PPIs between the TLS proteins play a crucial role in localizing the proper TLS DNA polymerase to the lesion site and in the polymerase switching events between the replicative and TLS polymerases [29,30,31,32]. Each TLS polymerase contains multiple domains that coordinate formation of the complex, recruitment to the damage site, and insertion/extension of the nascent DNA (Figure 2). The biochemical and structural characteristics of the individual PPIs within the TLS heteroprotein complex are critical factors affecting TLS regulation. For this review, we analyzed the available crystallographic and NMR structures of key TLS PPIs with a primary focus on PPIs that could potentially be disrupted by small molecules. For each of these TLS PPIs, we highlight the key intermolecular interactions that govern their affinity. We have extensively collated the number of hydrogen bonds, salt bridges, disulfide bonds, pi–pi stacking interactions, and van der Waals clashes for each PPI.

The formation of a protein–protein complex in aqueous solution at room temperature is a thermodynamic driven process. Non-bonded van der Waals and electrostatic interactions between the solute and the solvent that are lost in the process of complex formation are mostly compensated for by newly formed interactions at the protein–protein interface, leading to a change in the free energy of the system [33]. It has been observed that burying hydrophobic portions of the surface at the new protein–protein interface brings about a larger free energy gain than burying polar portions of the surface area [34,35]. Herein, we have calculated the van der Waals surface complementarity and buried solvent-accessible surface area (SASA) for the analyzed TLS PPIs [36]. Key interactions at the various TLS PPI interfaces are described throughout the text with detailed descriptions of all the intermolecular interactions, and the energy terms that govern these TLS PPIs are provided in the Supplementary Tables.

### 2.1. PCNA PPIs

**Figure 3:**
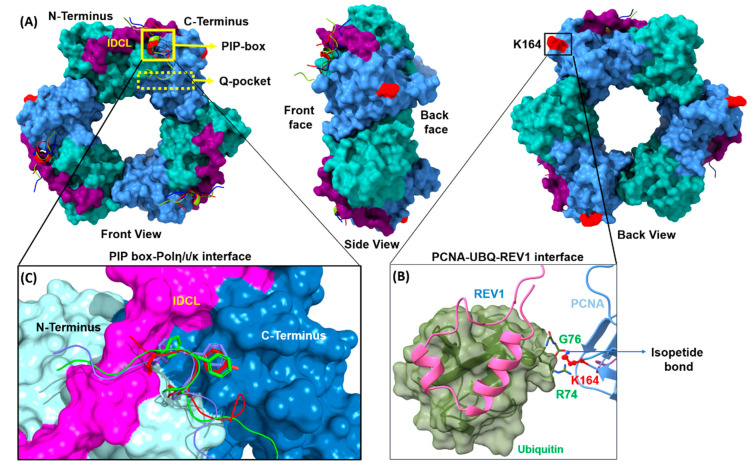
Trimeric structure of PCNA. (**A**) Structure of human PCNA complex with POLη (PDB ID 2ZVK), POLι (PDB ID 2ZVM), POLκ (PDB ID 2ZVL) highlighting the homotrimer subunits with C-terminus, N-terminus and IDCL colored in blue, green and purple, respectively. (**B**) Magnification of the monoubiquitinated PCNA (PDB ID 3TBL) aligned with the ubiquitin-REV1 complex (PDB ID 6ASR). (**C**) Closer images of the PIP-box motifs of the inserter polymerases bound PCNA.

In human cells, PCNA plays a critical role in anchoring all the TLS polymerases to DNA and modulating the exchange process during DNA replication and repair. In the TLS complex, PCNA is a homotrimer comprised of three individual subunits linked by an interdomain connector loop (IDCL) (Figure 3). The homotrimer is assembled as a circular ring with an open central region wide enough to encircle DNA and to allow diffusion of PCNA along the DNA strand. The PCNA ring has one side facing the direction of DNA synthesis and the other side pointing away. The “front” side of the PCNA complex faces towards the DNA and contains the C-terminus of each monomer and the IDCL. The internal surface of PCNA is positively charged, which allows it to clamp around the negatively charged DNA, whereas the outer surface has several β-sheets adjacent to the C-terminal loop capable of interacting with multiple motifs. PCNA can directly interact with all other TLS polymerase through a series of PPIs.

The C-terminal domain of PCNA plays an important role in the initiation of TLS. In response to DNA damage, a ubiquitin moiety (76aa) is covalently attached to the conserved K164 of PCNA via an isopeptide bond. Incorporation of the ubiquitin serves as the initial step in recruitment of the TLS polymerases. This isopeptide bond formation is primarily facilitated through an enzyme cascade of the E1 ubiquitin-activating enzyme Ube1 and a complex comprised of Rad6 (E2 ubiquitin-conjugating enzymes) and Rad18 (E3 ubiquitin ligase). Addition of the ubiquitin to PCNA enhances its affinity for the ubiquitin-binding domains (UBM) of the Y-family of TLS polymerases. PCNA monoubiquitination is a reversible, dynamic modification. Removal of the ubiquitin is facilitated by ISGylation of PCNA with the ubiquitin-like protein ISG15 at K164 and K168, which ultimately terminates error-prone TLS to prevent excessive mutagenesis [37].

#### 2.1.1. PCNA/UBM and PCNA/UBZ PPIs

**Figure 4:**
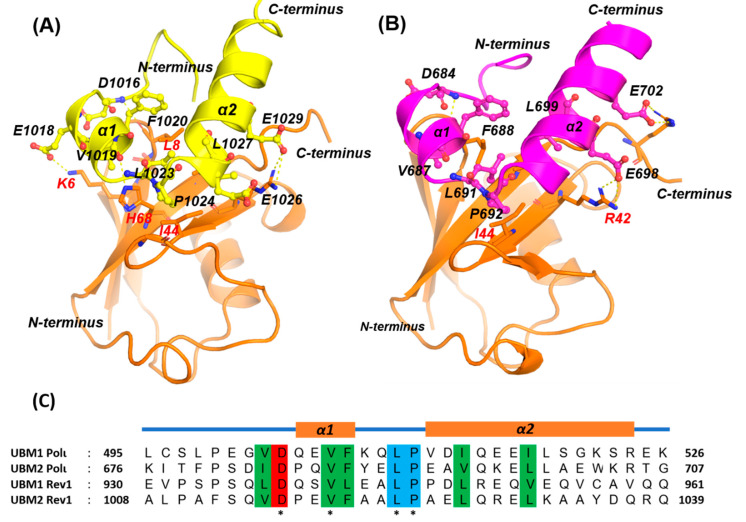
REV1/UBM complex structure. Structure of ubiquitin complex with UBM2 of REV1 (PDB ID 5VZM) (**A**) and POLι (PDB ID 2KTF) (**B**). Selected amino acid side chains of REV1 and POLι at the binding interface are shown in ball stick representation and labeled. (**C**) Sequence alignment of UBMs. Conserved residues are highlighted, with the signature “Leu-Pro” motif in blue, hydrophobic residues in green and negatively charged polar residues in red.

The monoubiquitination of PCNA at K164 promotes its binding to the ubiquitin-binding motifs (UBMs) of REV1 [19] and POLι [38] or the ubiquitin-binding zinc fingers (UBZs) of POLη [39] and POLκ [40] While REV1 contains two UBMs, the general consensus is that only the C-terminal UBM (UBM2) is capable of directly binding to ubiquitin [18,41]. POLι also contains two UBMs; however, both of the POLι-UBMs are capable of binding to UbPCNA. Both UBMs of POLι and REV1 consist of a well-conserved helix-turn-helix motif and adjacent folded stretches on both sides. The overall domain size is 30 residues and, upon identification, constituted a new type of α1-α2 fold. NMR solution structures of the UBMs of REV1 and POLι in complex with UbPCNA demonstrate a very similar ubiquitin-binding mode with the PPI interface centered on the L8 residue of ubiquitin [18,19,38,41,42] The UBMs bind to ubiquitin on the hydrophobic surface delineated by L8, I44, and V70. Key interacting residues in the structure of REV1-UBM2 bound to ubiquitin are conserved in the POLι UBM2–ubiquitin structure and are involved in the same hydrophobic intermolecular contacts in both complexes. These conserved residues are as follows for REV1/POLι: V1019/V687, F1020/F688, L1023/L691, P1024/P692, L1027/V695, and E1030/E698. The conserved N-terminus of both REV1 and POLι, D1016–V1019/D684–V687, are stabilized by hydrophobic interactions with the K6, K8 and T9 residues of ubiquitin. The difference between the two complexes is most pronounced toward the C-terminal end of the second α-helix of UBM2, including residues L699 and W703 in POLι, corresponding to L1031 and Y1035 in REV1, and contact residue L73 at the C-terminus of ubiquitin (Figure 4). In addition, UBM2 also use electrostatic interactions by forming a salt bridge between R-42 of ubiquitine and conserved E1030/E698 glutamate residues in REV1/ POLι.

#### 2.1.2. PCNA/PIP-Box PPIs

**Figure 5:**
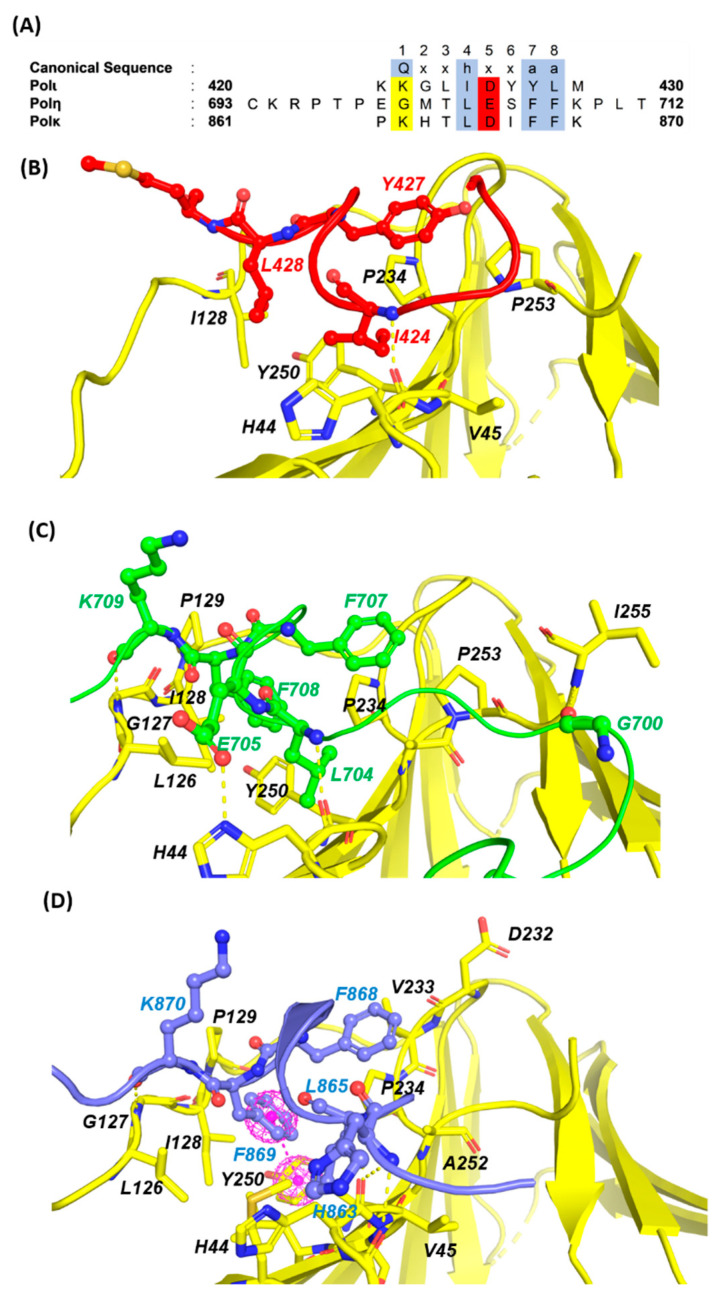
PCNA/PIP-box interactions. (**A**) The eight residues of canonical and non-canonical PIP-boxes of the inserter polymerases. The canonical PIP-box residues at positions 1, 4, 7, and 8 are highlighted in blue, acidic residues conserved at position 5 are highlighted in red, and basic residues are highlighted in yellow. Crystal structures of the human PIP-box motifs from POLι ((**B**), red), POLη ((**C**), green), and POLκ ((**D**), blue) complexed with PCNA (yellow). Select amino acid side chains at the binding interface are labeled.

The homotrimeric PCNA complex also contains three identical hydrophobic PCNA Interacting Protein (PIP) binding sites located adjacent to the interdomain-connecting loop on the “front” side of each monomer. Adjacent to the primary hydrophobic PIP-box binding site on PCNA is a smaller region termed the “Q” pocket in which the side chain of the initial Q residue contained in canonical PIP-box motifs forms hydrogen bonds with several PCNA residues. These binding sites serve as the primary platform by which PCNA interacts with the inserter TLS polymerases through conserved PIP-box motifs, which typically contain eight residues. Structural studies demonstrate that POLη (701-710), POLι (421–428), and POLκ (701–708) bind PCNA through a highly divergent PIP-box lacking the strict consensus PIP-box sequence *Qxxhxxaa*, where *h* is a hydrophobic residue, *a* is an aromatic residue, and *x* is any residue (Figure 5) [43,44]. As such, the PIP-box motifs contained in the TLS DNA polymerases are considered non-canonical and comprised of the following sequence: (K/G)xx(I/L/)xxFF(Y/L) To date, almost 80 PIP-box containing proteins have been described as interacting with PCNA in the literature. The molecular recognition of PCNA and the Y-family of polymerases has been characterized by an integrative structural approach and shows a unique mode of binding to PCNA that extends outside the canonical PIP-box on PCNA.

Despite none of the inserter polymerases containing the initial glutamine residue and POLι not containing the first aromatic residue, all three Y-family pols are stabilized by adopting the characteristic PIP-box 3_10_ helix conformation. The hydrophobic trident residues, hxxaa, insert into the hydrophobic PIP pocket of PCNA. The central segment of each POL where the PIP box is located, binds tightly to the front and inner sides of the ring, while the N- and C-terminal tails remain disordered at opposite sides of the ring. Our sequence alignment analysis of the non-canonical PIP boxes of Y-family POLs revealed that the presence of an acidic residue at position 5 appears to stabilize the 3_10_ helix and might be required for a high affinity interaction (Figure 5). The structural alignment also revealed that the side chain of M701 and T702 of POLη intrudes into the adjacent Q-pocket and forms hydrophobic interactions with V45, A208, Y211 and L251. By contrast, K862 of POLκ and K421 of POLι are highly disordered and incapable of interacting with the Q-pocket. The canonical PIP-box sequence (hxxaa) residues of all the y-family POLs are very similar to each other and form a hydrophobic 3_10_ helix plug, which fits into a hydrophobic pocket comprising of M40,V45, S46, L47, I128, P234, Y250, and P252 residues of PCNA.

As noted above, POLη and POLκ have one and two UBZ domains, respectively, at the N-terminal side of their PIP-box motif, whereas POLι has two UBMs at the C-terminal side of its PIP-box motif (Figure 2). As the atomic distance between the UBZ domains and PIP-box are minimal (~40 residues in POLη and ~60 in POLκ), the UBZs are likely to interact with the ubiquitin moiety linked to K164 within the same subunit of trimeric PCNA. By contrast, the distance between the UBM and PIP-box (~250 residues) of POLι enable the proximal UBM domain to be positioned properly to interact with the ubiquitin moiety linked to K164 with the subunit bound to the PIP-box. This may possibly explain why POLι can independently function as a TLS polymerase by directly interacting with PCNA via the UBM domain and the PIP domain.

### 2.2. REV1 PPIs

REV1 plays important structural and regulatory roles in TLS, with its primary function to serve as a scaffold for assembly of the multiprotein TLS complex and regulate recruitment of other TLS polymerases. As noted above, initial recruitment of REV1 to the stalled replication fork is mediated via two PPIs with UbPCNA. REV1 is also responsible for the recruitment of the inserter and extender polymerases to the lesion site through specific PPIs with these polymerases. These are localized to the C-terminal domain of REV1 (REV1-CT, residues 1157–1251), which can bind REV1 interacting regions (RIRs) from the Y-family polymerases POLη, POLι, and POLκ and the POLD3 subunit of the B-family polymerase POLζ on one face, while simultaneously binding the regulatory subunit REV7 of POLζ on the opposite face. The REV1-CT/RIR PPI is discussed in this section. Because the REV1-CT/REV7 PPI is inducible only after several PPIs of subunits in POLζ, we have included the discussion of this essential REV1 PPI in the section below dedicated to POLζ.

#### REV1-CT/RIR PPI

**Figure 6:**
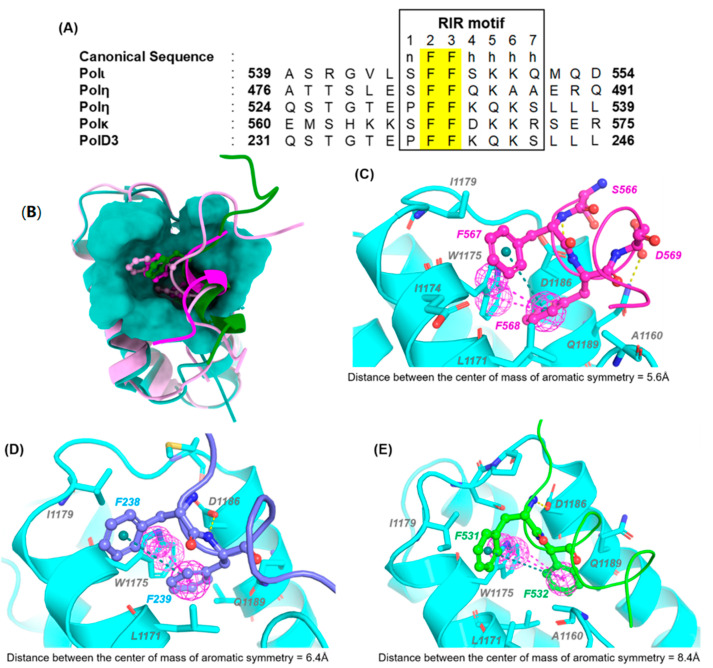
REV1-CT/RIR Structures and Interactions. (**A**) Aligned sequences of RIR motifs from various TLS polymerases. (**B**) Overlayed structures of the RIR motifs from POLη (green), POLκ (magenta), and POLD3 (pink) bound to REV1-CT. REV1-CT/RIR PPI interfaces and FF orientations for POLκ (**C**), POLD3 (**D**), and POLη (**E**).

Co-crystal and NMR structures of REV1-CT interacting with RIR motifs from POLD3 (2N1G) [45], POLη (2LSK) [46], and POLκ/REV7 (4GK5) [47] reveal the putative protein-interaction groove and the REV1-CT structural framework that allows it to selectively recognize RIR motifs. Within this complex, REV1-CT adopts a four-helix bundle structure comprised of α-helices α1 (1165–1178), α2 (1184–1199), α3 (1203–1219) and α4 (1224–1243) (Figure 6). The N-terminus of REV1-CT (residues 1157–1164) forms a rigid type I′ β-hairpin that is stabilized by two intermolecular hydrogen bonds between L1159 and A1162. The β-hairpin and the first two α-helices α1 and α2 in the N-terminus of REV1-CT interact with the RIR motifs.

The RIR motif is characterized by two phenylalanine residues preceded by an N-cap residue and followed by at least four helix forming residues (nFFhhhh, Figure 6). Multiple structural and mutational studies have clearly demonstrated that the FF residues interact with REV1-CT through strong hydrophobic interactions that are essential for REV1-CT/RIR binding [46,47]. The type I′ β-hairpin of REV1-CT is packed tightly against helices α1 and α2. In this region, the hydrophobic side chains of L1171 and W1175 (α1) and the negatively charged side chain of D1186 (α2) form a prominent pocket (393 Å^3^) on the surface of REV1-CT that interacts with both of the essential FF residues. The primary hydrophobic pocket is formed by side chains from L1159 (β-hairpin); L1171, L1172, and W1175 (α1); and D1186, Q1189, and V1190 (α2). The depth of this pocket allows for the complete insertion of one of the F residues from the RIR motif. The second F residue binds in a shallower binding groove adjacent to the primary binding pocket and forms key intermolecular interactions with E1174, W1175, and I1179, which are all contained in α1 (Figure 6). In addition to the hydrophobic interactions between the FF residues and REV1-CT, the complex is stabilized by polar interactions between the side chain of D1186 and the backbone amide between the FF motif. It is possible that the REV1-CT/RIR PPI is stabilized by the presence of several residues following the FF motif; however, the lack of any conserved residues in this region suggests that the putative stabilization does not depend critically on any specific side intermolecular interactions other than those formed by the two FF residues [48]. Although the mode of RIR interaction with REV1-CT is conserved across all three complexes, the orientation and the distance between the FF residues of POLη, POLκ and POLD3 differ slightly across the NMR and x-ray crystal complex. The orientation of the hydrophobic core of the RIR motif may affect the compactness of the FF residues inside the active side of REV1, which may explain the relative affinity POLη, POLκ and POLD3 with respect to REV1-CT.

### 2.3. POLζ PPIs

As noted above, the primary role of POLζ in TLS is to extend the primer DNA past the nucleotide inserted by one of the Y-family polymerases [49]. POLζ consists of several subunits: (1) REV3, the catalytic subunit; (2) REV7, an accessory subunit that serves as a bridge between REV3 and REV1; and (3) POLD2 and POLD3, subunits of the replicative polymerase POLδ that enhance the efficacy of POLζ-mediated TLS. REV3 is a member of the B-family DNA polymerase family, which also includes POLs α, δ and ε and serves as the catalytic subunit of POLζ [50]. REV7 is an adapter protein that mediates the essential TLS interactions between REV3 and REV1, thereby mediating the second polymerase switching in TLS [51]. The crystal structures of human POLζ (PDB IDs 3VU7 and 4KG5) complexed with REV1 reveals the mechanism underlying the REV1/POLζ complex formation [47,52]. A conformational change induced by the REV7/REV3 PPI unexpectedly provides an interface for binding of REV1-CT to REV7. These interactions are described in this section. In the presence of REV3, REV7 undergoes a structural rearrangement of the “safety belt region”, resulting in a complex in which REV7 is composed of three α-helices (αA,αB, and αC), eight β-strands (β2, β3, β4, β5, β6, β7, β8, and β8″), and four 3_10_ helices (3α-1, 3α-2 and 3α-3) [53]. This conformational change in the seatbelt of REV7 provides an interface for interaction with REV1-CT and enables formation of the complete REV1/POLζ complex.

#### 2.3.1. REV7/REV3 PPI

**Figure 7:**
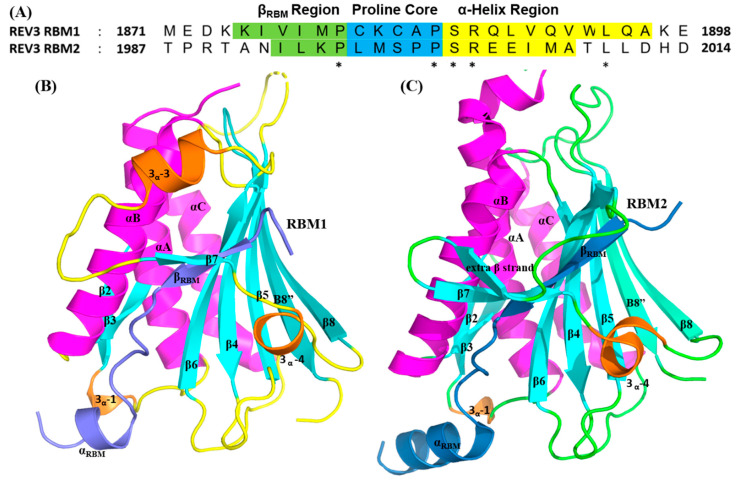
Structure of the REV7/REV3 complex. (**A**) Sequence alignment of RBM1/2 of REV3 and the residues that interact with the primary binding regions on REV7. Important binding regions are colored and conserved residues are denoted with an asterisk. Secondary structure comparisons of REV7 in complex with REV3_RBM1_ (**B**) and REV3_RBM2_ (**C**). The secondary structures of REV7 and the REV3 RBMs are shown by arrows and cylinders. For REV7, α-helices are magenta and β-strands are cyan. For REV3 RBMs, α-helices are orange and β-strands are blue.

Within the context of TLS, the REV7 subunit of POLζ serves to connect the inserter and extender polymerases by simultaneously binding to REV3 and REV1-CT. The C-terminal region of REV3 contains two adjacent, highly conserved REV7-binding motifs (RBMs) (RBM1, residues 1875-1896: RBM2, residues 1991–2012) that directly interact with REV7. These two RBMs contains a consensus sequence of ϕϕxPxxxxPSR, where ϕ represents an aliphatic residue and X represents any amino acid residue. Structurally, the most notable feature of this PPI is that a portion of REV3_RBM1_ and REV3_RBM2_ are inserted into the bridge-like safety belt structure of REV7, which results in homodimerization of REV7 through the canonical HORMA interface. As per the crystal structures of REV7/REV3_RBM1_ (PDB ID 3VU7) and REV7/REV3_RBM2_ (PDB code: 6B68), REV7 acts as an adapter protein to recruit REV7-binding motifs (RBMs) of REV3 (RBM1, residues 1875–1896: RBM2, residues 1991–2012). When complexed with REV3_RBM1_, REV7 adopts the similar secondary structures described above. By contrast, in the REV3_RBM2_/REV7 complex, the 3α-2 helix is not formed and an additional β-strand leading into a β-turn is formed with the β7 sheet (Figure 7).

The crystal structures of REV7 bound to the individual REV3 RBMs revealed that the key interacting residues of REV7 that bind to both REV3_RBM1_ and REV3_RBM2_ are conserved and are involved in the same van der Waal and electrostatic contacts in the each complex (Figure 7). REV3_RBM1_ and REV3_RBM2_ are comprised of three primary regions, which we define as follows: (1) β_RBM1_ (REV3_RBM1:_I1877–P1880/REV3_RBM2:_K1991–P1996), which forms an anti-parallel β-sheet comprised of β6 and β7′, (2) the proline core region (REV3_RBM1:_L1881–P1885/REV3_RBM2:_C1997–P2001), and (3) an α-helix binding region (REV3_RBM1:_S1886–A1892/REV3_RBM2:_S2002–A2012). Residues of the REV3 β_RBM1_ regions form a network of hydrogen bonds with β6 and β7′ of REV7. In addition to the hydrogen bonds in the β-sheet, the side chain phenols of tyrosine residues Y37 and Y63 of REV7 interact with the carbonyl oxygen atoms of the highly conserved P1885/P2001 and M1882, respectively. The proline core residues of both REV3_RBM1_ and REV3_RBM2_ are accommodated inside a large hydrophobic pocket formed by the aromatic side chains of Y37, Y63, and F146 of REV7. The less-conserved C-terminal α-helix region of REV3_RBM1_ or REV3_RBM2_ adopt distinct binding modes with REV7. I1890 (REV3_RBM1_) or V2006 (REV3_RBM1_) act as an anchor in the α-helix binding sequence and is essential for high-affinity REV7 binding. For REV3_RBM1_, W2009 provides an additional anchoring surface to improve its affinity for REV7. Dimerization of REV7 appears to contribute to the proper biological function of POLζ in mammalian cells [53]; however, a structure of the dimer has not been determined and the exact interaction site(s) have yet to be identified.

#### 2.3.2. REV1-CT/ REV7 PPI

**Figure 8:**
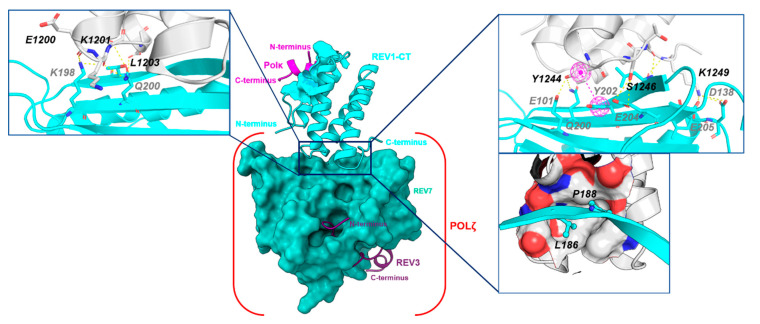
REV1-CT/REV7 PPI interface. The POLκ-RIR/REV1-CT/REV7/REV3_RBM1_ multi-protein complex demonstrates key interactions at the REV1-CT/REV7 interface.

As noted above, the PPI between REV7 and REV3 induces a conformational change in REV7, which promotes binding to REV1-CT. The co-crystal structure of REV1-CT complexed with REV7 (PDB ID 4GK5) demonstrates that the C-terminal tail and the α2-α3 loop of REV1-CT interacts with the C-terminal β-sheet of REV7 (β8′ and β8″), residues of β5 (E101), and the αC-β6 loop (L137 and D138) to form the REV1-REV7 PPI interface (Figure 8) [47,54]. At the REV1-CT/REV7 interface, the side chains of Y1244, S1246, and K1249 (REV1-CT) interact with the side chains of Q200 (β8″), E101 (β5), E204 (β8″), E205 (β8″) and D138 (αC-β6) of REV7 through several hydrogen bonds. REV1-CT residues T1247 and K1249 form additional hydrogen bonds with L186 on REV7 to enhance the binding interaction. In the REV1-CT α2-α3 loop, the side chains of D1202 and E1204 form salt bridges with T191 and K190, respectively, on the β8′ sheet of Rev7. The amide backbones of REV1-CT residues E1200 and K1201 also form hydrogen bonds with K198 and Q200, respectively, on the β8″ sheet of Rev7. In addition to these hydrophilic interactions, REV1-CT residues L1203, Y1244, and L1248 of REV1-CT form a “hydrophobic cage” that accommodates the side chains of L186 and P188 of REV7. In this pocket, the side chain of Y1244 forms a strong pi–pi interaction with the phenyl ring of Y202 (β8″ on REV7). Mutational studies for REV1-CT at the REV7 interface clearly demonstrate that the hydrophobic interactions contribute more significantly to the PPI than the electrostatic interactions [48].

## 3. Targeting TLS PPIs with Small Molecules

Small molecule inhibitors of the TLS PPIs are promising candidates for cancer therapeutics and also have potential as chemical probes to decipher the various roles of each polymerase in REV1/POLζ-dependent TLS. Compounds that disrupt TLS PPIs could serve in combination therapies to improve first-line chemotherapy as they can enhance the killing of cancer cells, while at the same time reducing the possibility of relapse and acquired chemoresistance by decreasing tumor mutation rate. Generally, PPIs occur through relatively large contact surfaces (1500 to 3000 Å^2^) [55], which makes disrupting them with a small molecule difficult because compounds that target these PPIs through competitive binding must typically have a high molecular weight to overcome the distributed free energy (ΔG) of a large PPI interface [56]. Extensive studies on PPIs have demonstrated that many contain a small number of “hotspot” residues, which contribute a disproportionate amount of the binding energy to the PPI [57,58].

With regard to the essential PPIs involved in the REV1/POLζ-dependent TLS complex, the average contact area size is considerably smaller (300 and 1000 Å^2^, Table 1), suggesting these may be particularly amenable to disruption with small molecules. For example, the overall surface area of the REV1-CT/RIR interface is 446 Å^2^ and the FF residues in the RIR motif are known hotspot residues that interact with a preformed binding pocket on REV1-CT. As such, it has been an attractive target for the development of small molecule TLS inhibitors. The molecular surface area of the PCNA/PIP-box PPI is in a comparable range (451–531 Å^2^, depending on the specific structure used for analysis). Each PIP-box motif from an inserter polymerase contains a series of five hydrophobic residues that insert into the hydrophobic PIP pocket of PCNA and provide essential interactions. Not surprisingly, this PPI has also been successfully targeted with small molecules. Other TLS PPIs are comprised of a larger molecular surface area or consist of primarily surface contacts, which may make them less amenable to small molecule disruption.

**Figure 9:**
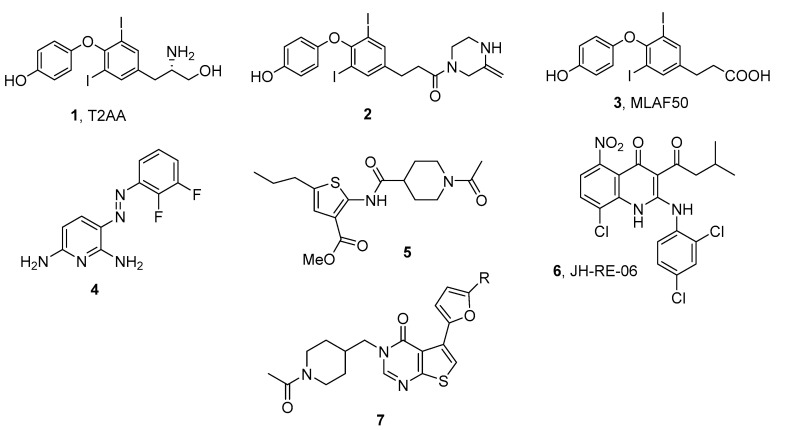
Structures of small molecules that disrupt TLS PPIs.

During the last several years, the identification and preliminary development of small molecules that disrupt several TLS PPIs have been reported. These compounds target multiple TLS PPIs at various levels of the TLS process. In general, these compounds are still at the early stages of drug development and demonstrate only modest in vitro activity. To date, only one of these compounds (JH-RE-06) has advanced into an in vivo model of human cancer. Structures and key data for these compounds are provided in Figure 9 and Table 2. More detailed information about the identification and development of these compounds has been reviewed elsewhere [60]. Structures of the following compounds complexed to their TLS protein target are available in the RCSB database: compound **1**, PDB ID 3WGW [61]; compound **4**, PDB ID 6WS0 [62]; compound **6**, PDB ID 6C8C [63].

## 4. Discussion

The importance of understanding the mechanisms that regulate error-prone TLS is highlighted by its implications for human health, particularly with respect to cancer prevention and treatment. As PPIs are essential for proper TLS-mediated replication, small molecules that target these interaction interfaces are promising candidates for cancer therapeutics. A key goal for the continued development of these compounds is to more clearly determine which TLS PPIs are druggable. The surface area analysis in Table 1 along with the development of small molecules targeting several of these TLS PPIs provides some insight into the characteristics needed for a small molecule to bind with a TLS PPI partner and disrupt a key interaction.

An additional line of study that has been minimally explored relates to the potential overlapping structural properties of PIP-box and RIR motifs. While the traditional paradigm has been that these motifs are distinct entities specific for a single target protein, recent studies have demonstrated clear sequence and structural similarities between PIP-box and RIR motifs [70]. One can envision the possibility through which a small molecule developed to target the PIP-box binding site on PCNA may also exhibit affinity for the RIR binding region on REV1-CT and vice versa. Based on the known biology of TLS, it is not clear whether a single compound targeting both PPIs would be a suitable strategy for inhibiting TLS. The percent of DNA damage in replicating cells that is bypassed in a REV1-dependent versus REV1-independent manner is not fully understood; therefore, a compound that inhibits TLS through disruption of a REV1 PPI may not fully inhibit cellular TLS. A single compound that targets multiple PPIs or a combination regimen of small molecules that target distinct TLS PPIs in the heteroprotein complex may be necessary for optimal inhibition of TLS.

The majority of compounds shown in Figure 7 were identified through de novo biochemical screens against the PPI. There are several additional strategies that could be utilized to identify small molecule inhibitors of TLS PPIs. Computational screens (docking- or pharmacophore-based) are an efficient way of evaluating large chemical libraries for compounds that can potentially disrupt the PPI. Several TLS PPIs lack substantial grooves or clearly defined pockets at their interface. As such, designing small molecule libraries for biochemical and computational screening that maximize the topological complexity of the library members to more closely resemble the PPI interface would enrich the libraries for compounds capable of disrupting PPIs. In addition, fragment-based screening by mass spectrometry, crystallography, or NMR could be utilized to target PPIs with a less-defined interface. Rational design of small molecules to mimic key structures essential for the PPI is also a promising strategy that has been underexplored with respect to TLS. Cyclic peptides, peptidomimetics, and stapled peptides are all being increasingly used to inhibit PPIs. A primary example of this for TLS was the identification of 4, which mimics the FF residue of the RIR motif to inhibit the REV1-CT/RIR PPI [66]. These type of ligands mimic natural protein–protein contacts by presenting multiple amino acid side chains from architecturally complex cores. Finally, small molecule–protein hybrids have been developed to artificially increase apparent molecular mass and target the most difficult PPIs [71,72,73,74].

Other than directly targeting PPIs essential to the TLS heteroprotein complex, there are several other approaches to identify and develop TLS inhibitors. As ubiquitination is the first step in TLS, preventing or reversing ubiquitination at K164 may prevent TLS initiation. Although enzymes involved in ubiquitination have been targeted with small molecules, it is unlikely that this approach will be selective for inhibiting TLS initiation. Neddylation and isgylation at K164 antagonize the initial ubiquitination step preventing the recruitment of TLS POLs; therefore, the ability to stimulate these modifications to PCNA could also prevent TLS initiation. Preventing the catalytic activity of the inserter or extender polymerases could also result in TLS inhibitors; however, this strategy is likely to provide non-specific inhibitors of multiple polymerases, including replicative polymerases.

## Figures and Tables

**Table 1:** Analysis of potential binding pockets at the interface of TLS PPIs.

PPI ^a^	Protein/Region	Molecular Surface Area (Å^2^) ^b^	Hydrophobic Surface Area (Å^2^) ^b^	Hydrophilic Surface Area (Å^2^) ^b^
REV1-CT/RIR (PDB ID: 4GK5)	FF binding pocket	446.4	243.7	202.7
REV1-CT/REV7 (PDB ID 3VU7)	C-terminal tail α2-α3	265.3 232.7	118.0 ---	147.3 232.7
REV7/REV3_RBM1_ (PDB ID:3VU7)	β_RBM1_ proline core α-helix region	748.6 380.2 551.3	365.3 238.2 247.4	383.3 142.0 303.9
REV7/REV3_RBM2_ (PDB ID: 6BC8)	β_RBM1_ proline core α-helix region	1044.0 352.8 644.6	578.1 184.1 247.6	465.9 168.7 397.0
PCNA/PIP-box (PDB ID: 2ZVK)	Hydrophobic pocket Q-pocket	451.4 385.2	212.3 219.9	239.1 165.3
PCNA/PIP-box (PDB ID: 2ZVM)	Hydrophobic pocket Q-pocket	486.0 283.6	241.6 183.6	244.4 100.0
PCNA/PIP-box (PDB ID: 2ZVL)	Hydrophobic pocket Q-pocket	531.9 283.6	329.7 183.6	202.2 100.0
UbPCNA/UBM-POLι (PDB ID: 2KTF)	α1-α2 region	893.4	338.3	555.1
UbPCNA/UBM-REV1 (PDB ID: 5VZM)	α1-α2 region	860.0	355.4	504.6

^a^ The underlined protein contains the binding pocket analyzed for surface area. ^b^ Surface areas were calculated in BioLuminate (Schrödinger, 2021) using a solvent probe radius of 1.4 Å [59].

**Table 2:** Key early stage data for small molecule inhibitors of TLS PPIs.

Compound	TLS PPI Target	PPI Disruption IC_50_ (µM)	References
**1**, T2AA	PCNA/PIP-box	~1.5	[64]
**2**	UbPCNA/REV1	3.4	[65]
**3**, MLAF50	Ub/REV1-UBM2	176	[19]
**4**	REV1-CT/RIR	0.99	[66]
**5**	REV1-CT/RIR	4.1	[67,68]
**6**, JH-RE-06	REV1-CT/REV7	0.78	[63]
**7**	REV7/REV3	78	[69]

## Supplementary Materials

The following are available online, Tables describing key interactions and data for each TLS PPI; surface complementarity algorithm.
