# Supplementary material for: Protein–Protein Interactions in Translesion Synthesis
Source: Molecules. 2021 Sep 13;26(18):5544. doi: 10.3390/molecules26185544 (PMC8468184; doi:10.3390/molecules26185544)
Supplement: Supplementary file 1 [file molecules-26-05544-s001.zip › molecules-1360983-supplementary.pdf]

molecules

Supplementary Materials

Protein–Protein Interactions in Translesion Synthesis

Radha Charan Dash and Kyle Hadden *

Department of Pharmaceutical Sciences, University of Connecticut, 69 North Eagleville Rd, Storrs, CT 06029-3092, USA; radha.dash@uconn.edu
* Correspondence: kyle.hadden@uconn.edu; Tel.: +1-860-486-8446

Polι-UBQ					
**Polι**	**UBQ**	**Distance**	**Specific Interactions**	**Surface Complementarity**	**Buried SASA**
F679	L73	4.0 A		0.68	9.40%
S681	T9	3.1 A		0.89	12.80%
D682	T9	2.9 A		0.69	79.60%
	L8	3.3 A			
	I36	3.5 A			
	T7	3.6 A			
	L69	3.9 A			
I683	L8	3.7 A		0.77	85.30%
	T9	3.9 A			
D684	L8	2.9 A	H-Bond with L8	0.85	46.80%
	T9	3.4 A			
V687	H68	3.5 A		0.87	99.60%
	L8	3.5 A			
	G10	3.5 A			
	K6	3.8 A			
	t7	3.8 A			
F688	L8	3.6 A		0.95	9.00%
E690	H68	3.4 A		0.93	21.90%
L691	V70	3.4 A		0.89	91.10%
	L8	3.8 A			
	H68	4.0 A			
P692	G47	3.5 A		0.86	71.60%
	I44	3.7 A			
V695	Q49	3.3 A		0.89	87.60%
	I44	3.8 A			
E698	R42	2.8 A	Salt bridge with R42	0.63	35.20%
L699	L71	3.5 A		0.82	99.30%
	V70	3.6 A			
E702	R74	2.8 A	Salt bridge with R74	0.89	68.10%
	L73	3.4 A			
W703	L71	3.5 A		0.79	70.40%
	L73	3.5 A			
T706	L73	3.4 A		0.85	30.90%
**REV1-UBQ**					
**REV1**	**UBQ**	**Distance**	**Specific Interactions**	**Surface Complementarity**	**Buried SASA**

P1010	L8	3.7 A		0.84	69.70%
Q1014	T9	3.4 A		0.95	24.70%
V1015	L8	3.4 A		0.86	42.30%
D1016	L8	2.9 A	H-Bond with L8	0.85	44.20%
	G10	3.7 A			
	T9	3.7 A			
E1018	L6	2.9 A	H-Bond with K6	0.61	18.80%
V1019	H68	3.0 A	H-Bond with H68	0.85	98.40%
	L8	3.2 A			
	T7	3.6 A			
	L6	3.8 A			
F1020				0.74	5.30%
A1022	H68	3.8 A		0.81	27.70%
L1023	H68	3.5 A		0.77	99.90%
P1024	G47	3.4 A		0.9	75.80%
	I44	3.7 A			
L1027	I44	3.3 A		0.77	100.00%
	R42	3.6 A			
	V70	3.8 A			
E1030	R42	2.8 A	Hbond with R42	0.85	51.20%
	L73	3.8 A			
L1031	L73	3.7 A		0.8	99.90%
	L8	3.8 A			
A1034	L73	3.9 A		0.78	58.40%
**PCNA-Polι PIP-Box**					
**Polι**	**PCNA**	**Distance**	**Specific Interactions**	**Surface Complementarity**	**Buried SASA**
K421	I255	3.3 A		0.93	27.20%
G422	A252	3.4 A		0.94	79.70%
	I255	3.6 A			
	P253	3.7 A			
	V45	3.7 A			
L423	S43	3.5 A		0.9	40.60%
	H44	3.5 A			
	A252	3.9 A			
I424	H44	2.8 A	H-Bond with H44	0.88	99.10%
	V45	3.4 A			
	A252	3.6 A			
	M40	3.7 A			
	P234	3.8 A			
	S46	3.8 A			
	L47	3.9 A			
	Y250	4.0 A			
D425	H44	3.4 A		0.91	48.60%
	M40	3.5 A			
Y427	P253	3.4 A		0.82	82.70%
	D232	3.5 A			
	P129	3.5 A			
	P234	3.6 A			

L428	G127<br>L126	3.7 A<br>4.0 A		0.75	81.60%
M329	G127<br>L126	2.8 A<br>3.9 A	H-Bond with G127	0.79	31.00%
**PCNA-Polη PIP-Box**					
**Polη**	**PCNA**	**Distance**	**Specific Interactions**	**Surface Complementarity**	**Buried SASA**
P698	Y211<br>S43	3.4 A<br>3.8 A		0.87	67.90%
E699	K254	3.7 A		0.71	10.70%
G700	I255<br>K254	2.7 A<br>3.6 A	H-Bond with I255	0.7	56.00%
M701	A252<br>V45<br>A208<br>L251	3.1 A<br>3.6 A<br>3.8 A<br>3.9 A		0.87	87.60%
Q702	P253<br>A252<br>I254<br>V45	2.9 A<br>3.2 A<br>3.5 A<br>3.6 A		0.86	47.50%
T703	H44	3.4 A		0.79	38.90%
L704	H44<br>S46<br>L47<br>M40<br>V45<br>P234<br>A252<br>L126	2.9 A<br>3.4 A<br>3.5 A<br>3.5 A<br>3.6 A<br>3.7 A<br>3.7 A<br>3.9 A	H-Bond with H44	0.9	100.00%
E705	H44<br>E124	2.7 A<br>3.2 A	H-Bond with H44	0.85	51.80%
F707	D232<br>P234<br>P253<br>A252	3.4 A<br>3.6 A<br>3.6 A<br>4.0 A		0.89	57.00%
F708	Y250<br>I128<br>L126<br>G127<br>P129<br>P234	3.5 A<br>3.5 A<br>3.7 A<br>3.7 A<br>3.9 A<br>4.0 A		0.87	97.40%
K709	G127<br>L126	3.1 A<br>4.0 A	H-Bond with G127	0.8	7.60%
P710	125<br>L126	3.3 A<br>3.7 A		0.85	48.20%
L711	Q125	2.9 A	H-Bond with G127	0.77	36.00%
**PCNA-Polκ PIP-Box**					
**Polκ**	**PCNA**	**Distance**	**Specific Interactions**	**Surface Complementarity**	**Buried SASA**
P861	V45	3.3 A		0.85	28.00%

K862	S43	3.3 A		0.85	21.20%
H863	S43<br>H44	3.1 A<br>3.2 A	H-Bond with S43<br>H-Bond with H44	0.88	30.50%
T864	A252<br>H44<br>V45	3.2 A<br>3.5 A<br>3.9 A		0.86	79.70%
L865	H44<br>S46<br>P234<br>L47<br>M40<br>V45<br>A252	3.2 A<br>3.3 A<br>3.5 A<br>3.5 A<br>3.5 A<br>3.6 A<br>3.9 A	H-Bond with H44	0.88	100.00%
D866	M40<br>H44	3.4 A<br>3.5 A		0.77	39.30%
F868	D232<br>P234<br>V233	3.1 A<br>3.6 A<br>4.0 A		0.86	56.00%
F869	G127<br>L126<br>L47<br>Y250<br>I128<br>P129	3.3 A<br>3.5 A<br>3.5 A<br>3.6 A<br>3.9 A<br>4.0 A	Pi–pi stack to Y250	0.81	95.00%
K870	G127<br>L126	3.2 A<br>3.7 A	H-Bond with G127	0.87	9.30%
**REV1-CT/Polκ RIR**					
**Polκ**	**REV1**	**Distance**	**Specific Interactions**	**Surface Complementarity**	**Buried SASA**
S566	M1183<br>D1186<br>E1185	3.2 A<br>3.2 A<br>3.3 A		0.86	66.30%
F567	D1186<br>W1185<br>L1171<br>I1179	3.1 A<br>3.3 A<br>3.6 A<br>3.7 A	H-Bond with D1186	0.86	90.70%
F568	D1186<br>A1160<br>W1185<br>L1171<br>V1190	2.7 A<br>3.5 A<br>3.5 A<br>3.7 A<br>3.7 A	H-Bond with D1186	0.82	99.30%
D569	Q1189	3.4 A		0.25	28.00%
K571	A1160<br>L1171	3.2 A<br>3.5 A		0.68	44.30%
**REV1-CT/Polη RIR**					
**Polη**	**REV1**	**Distance**	**Specific Interactions**	**Surface Complementarity**	**Buried SASA**
G529	P1182	3.1 A		0.45	24.30%
P530	P1182<br>D1186	3.6 A<br>3.6 A		0.75	49.50%

F531	D1186<br>P1182<br>W1175<br>I1179<br>D1181	3.1 A<br>3.4 A<br>3.5 A<br>3.7 A<br>4.0 A	H-Bond with D1186	0.79	70.50%
F532	D1186<br>L1159<br>A1160<br>Q1189<br>L1172<br>V1190<br>W1175	2.9 A<br>3.4 A<br>3.4 A<br>3.4 A<br>3.4 A<br>3.5 A<br>3.5 A	Pi–pi stack to W1175	0.89	99.80%
K533	D1186<br>Q1189	3.3 A<br>3.7 A	H-Bond to D1186	0.59	46.60%
K535	A1160<br>G1161	3.1 A<br>3.3 A		0.9	29.60%
S536	G1161<br>A1160	3.5 A<br>3.7 A		0.87	45.50%
L539	G1161<br>N1158	3.4 A<br>3.5 A		0.79	39.20%
**REV1-CT/PolD3 RIR**					
**PolD3**	**REV1**	**Distance**	**Specific Interactions**	**Surface Complementarity**	**Buried SASA**
N233	E1185<br>M1183	3.1 A<br>3.4 A	H-Bond with E1185	0.85	20.60%
M234	M1183<br>E1185	3.6 A<br>3.8 A		0.87	39.50%
M235	M1183	3.7 A		0.72	15.90%
S236	M1183	3.2 A		0.78	4.30%
N237	E1185<br>D1186<br>M1183	3.0 A<br>3.4 A<br>3.4 A		0.82	95.40%
F238	D1186<br>W1175<br>E1174<br>I1179<br>M1183<br>L1171	3.1 A<br>3.2 A<br>3.4 A<br>3.6 A<br>3.8 A<br>3.8 A	H-Bond with D1186	0.84	64.00%
F239	D1186<br>A1160<br>L1171<br>Q1189<br>V1190<br>W1175<br>L1172<br>L1159	3.0 A<br>3.3 A<br>3.3 A<br>3.4 A<br>3.4 A<br>3.4 A<br>3.6 A<br>3.8 A	H-Bond with D1186	0.83	100.00%
G240	D1186	3.7 A		0.77	21.40%
A242	A1160<br>L1171	3.4 A<br>3.9 A		0.69	41.80%
A243	A1160	3.5 A		0.67	37.50%

M244	A1160<br>G1161	2.9 A<br>3.7 A	H-Bond with A1160	0.89	28.00%
**REV1-REV7**					
**REV1**	**REV7**	**Distance**	**Specific Interactions**	**Surface Complementarity**	**Buried SASA**
E1200	K198	2.9 A		0.83	16.20%
E1201	Q200<br>K198<br>E101<br>T191	2.7 A<br>3.1 A<br>3.7 A<br>3.9 A	H-Bond with K198	0.83	52.30%
D1202	T191<br>Q200	3.4 A<br>3.4 A		0.9	57.40%
L1203	Q200<br>P188<br>Y202	2.9 A<br>3.3 A<br>4.0 A	H-Bond with Q200	0.77	100.00%
E1204	K190<br>T191<br>L189	3.3 A<br>3.3 A<br>3.9 A	H-Bond and salt bridge with K190	0.86	82.40%
D1207	P188	3.8 A		0.73	73.00%
L1240	L186	3.9 A		0.82	100.00%
Y1244	E101<br>Y202<br>Q200<br>E204	2.9 A<br>3.4 A<br>3.5 A<br>3.7 A	H-Bond with E101<br>Pi–pi stack to Y202	0.71	80.00%
G1245	P184	3.8 A		0.88	35.00%
S1246	E204<br>P184<br>L186	3.1 A<br>3.6 A<br>3.8 A		0.82	100.00%
T1247	L186<br>R185<br>P184	3.0 A<br>3.3 A<br>3.6 A	Hbond with L186	0.85	58.20%
L1248	L186<br>P188	3.5 A<br>3.9 A		0.83	85.30%
**REV7-REV3$_{RBM1}$**					
**REV3$_{RBM1}$**	**REV7**	**Distance**	**Specific Interactions**	**Surface Complementarity**	**Buried SASA**
T1874	A174	3.7 A		0.57	30.80%
A1875	L173<br>A155	3.5 A<br>3.6 A		0.83	44.60%
N1876	T152<br>H151<br>V150<br>E154<br>A155	2.9 A<br>3.4 A<br>3.6 A<br>3.8 A<br>4.0 A	H-Bond with T152	0.8	75.80%
I1877	A174<br>V150<br>L173<br>H151<br>V179	2.9 A<br>3.3 A<br>3.4 A<br>3.5 A<br>3.8 A	H-Bond with A174	0.82	95.40%

L1878	V150<br>I172<br>L173<br>L149<br>W171<br>M160	2.9 A<br>3.3 A<br>3.4 A<br>3.5 A<br>3.5 A<br>3.7 A	H-Bond with to V150	0.79	98.50%
K1879	I172<br>D178<br>W171<br>V148<br>L149<br>A174<br>T147	2.9 A<br>3.0 A<br>3.4 A<br>3.4 A<br>3.5 A<br>3.9 A<br>3.9 A	H-Bond with I172<br>Salt bridge to D178	0.83	80.40%
P1880	V148<br>W171<br>P170<br>Y63<br>F169	3.3 A<br>3.6 A<br>3.6 A<br>3.9 A<br>3.9 A		0.72	97.20%
L1881	P170	3.1 A	H-Bond with P170	0.81	44.60%
M1882	Y63<br>D168	2.9 A<br>3.6 A	H-Bond with Y63	0.56	42.20%
P1884	F146<br>Y63<br>Y37	3.2 A<br>3.3 A<br>3.9 A		0.88	92.60%
P1885	Y37<br>L60	2.4 A<br>3.8 A	H-Bond with Y37	0.72	91.70%
S1886	Y37	3.7 A		0.92	38.00%
R1887	V36<br>P38<br>Y37<br>E35	3.0 A<br>3.2 A<br>3.3 A<br>3.5 A	H-Bond with V36	0.88	63.60%
E1889	E59<br>E57	3.2 A<br>3.7 A		0.31	22.30%
I1890	E57<br>I41	3.2 A<br>3.9 A		0.63	95.70%
T1894	E57	2.9 A	H-Bond with E57	0.7	26.10%
**REV7-REV3RBM2**					
**REV3RBM2**	**REV7**	**Distance**	**Specific Interactions**	**Surface Complementarity**	**Buried SASA**
K1990	A156<br>A155	3.1 A<br>4.0 A	H-Bond with A156	0.71	15.00%
K1991	E81<br>E154<br>T152<br>R153	2.8 A<br>3.4 A<br>3.5 A<br>3.9 A	H-Bond and Salt bridge, to E81	0.89	54.50%
I1992	T152<br>E154<br>M160<br>L173	2.9 A<br>2.9 A<br>3.6 A<br>3.8 A	2 H-Bonds with T152<br>H-Bond with E154	0.84	88.30%

	A155	3.9 A			
	H151	4.0 A			
V1993	A174<br>V150<br>L173<br>H151	2.9 A<br>3.5 A<br>3.5 A<br>3.5 A	H-Bond with A174	0.9	88.10%
I1994	V150<br>I172<br>L149<br>I166<br>W171<br>L173	2.8 A<br>3.3 A<br>3.4 A<br>3.5 A<br>3.5 A<br>3.9 A	2 H-Bond with V150	0.85	97.60%
M1995	I172<br>D178<br>W171<br>V148<br>T147<br>A174<br>L149	2.8 A<br>3.3 A<br>3.3 A<br>3.4 A<br>3.4 A<br>3.8 A<br>3.9 A	2 H-Bond with I172	0.89	85.60%
P1996	V148<br>Y63<br>W171<br>P170	3.1 A<br>3.5 A<br>3.6 A<br>4.0 A		0.83	91.40%
C1997	P170<br>W171<br>I172<br>I163	3.0 A<br>3.6 A<br>3.8 A<br>3.8 A	1 H-Bond with P170	0.84	62.50%
K1998	Y63<br>E59	2.9 A<br>3.8 A	Salt bridge to E59<br>H-Bond with Y63	0.82	25.90%
C1999	Y63	3.5 A		0.89	17.40%
A2000	F146<br>Y37<br>Y63	3.3 A<br>3.4 A<br>3.4 A		0.88	90.80%
P2001	Y37<br>E59<br>L60	2.7 A<br>3.7 A<br>3.8 A	H-Bond with Y37	0.77	91.60%
R2003	E35<br>C144<br>V36<br>G143<br>Y37	2.9 A<br>2.9 A<br>3.2 A<br>4.0 A<br>4.0 A	2 H-Bond with E35<br>H-Bond with C144	0.81	75.00%
L1005	E59	3.5 A		0.72	23.00%
V2006	Y37<br>I41<br>H57<br>P38	3.7 A<br>3.7 A<br>3.8 A<br>3.9 A		0.88	100.00%
Q2007	P38	3.8 A		0.91	19.10%
W2009	C56<br>H57	3.5 A<br>3.5 A	2 pi–pi stack with H57	0.85	52.70%

	P58	3.7 A		
	I41	4.0 A		
L2010	G40	3.7 A	0.94	56.40%
	P38	3.8 A		

Surface Complementarity Algorithm:

The surface complementarity is calculated using the method of Lawrence and Colman [36]. The complementarity at any point on the molecular surface (Connolly surface) of one protein (A) is determined by finding the nearest point on the molecular surface of the other protein (B), and comparing the normal vectors to the two surfaces at those points, with a weighting function involving the distance between the points. Perfect complementarity is achieved if the vectors are parallel and the distance is zero. The function that defines the complementarity of A to B is:

$$S^{A\rightarrow B}(x_A) = (n_A.n'_A)exp[-w(|x_A - x'_A|)^2] \quad (1)$$

where x_A is the point on surface A, x'_A is the point on surface B nearest to x_A, n_A is the outward normal to surface A at x_A, n'_A is the *inward* normal to surface A at x'_A, and w is constant 0.5 Å^{-2}

The same is carried out for protein B, and the mean of the two median values is taken as the surface complementarity correlation,

$$S_c = (\{S^{A\rightarrow B}\} + \{S^{B\rightarrow A}\})/2 \quad (2)$$

where the braces represent the median value. A value of 1 means perfect matching of the surface shape; a value of 0 means that the shapes are uncorrelated. Further details about the limits on the surface and the treatment of buried surface regions can be found in [36] in the manuscript.
